# Neuroretinal damage associated with pituitary macroadenoma: endocrine and radiological predictors and correlation with optical coherence tomography-derived biomarkers

**DOI:** 10.3389/fendo.2026.1829465

**Published:** 2026-06-26

**Authors:** Diego Fernandez-Velasco, Elena Garcia-Martin, Belen Santamaria, María Jesús Rodrigo

**Affiliations:** 1 Hospital Universitario Miguel Servet, Servicio de Oftalmologia, Zaragoza, Spain; 2 Grupo de Investigación en Oftalmología Miguel Servet (GIMSO), Instituto de Investigación Sanitaria de Aragón (IIS Aragón), Universidad de Zaragoza, Zaragoza, Spain; 3 Universidad de Zaragoza, Zaragoza, Spain

**Keywords:** IGF-1, neuroretina, optical coherence tomography, pituitary macroadenoma, prolactin, visual field

## Abstract

**Objective:**

To identify endocrine and radiological tumor characteristics associated with higher risk of visual impairment.

**Methods:**

A retrospective observational study included 40 patients with pituitary macroadenoma. Variables analyzed were age, sex, disease duration, treatment, hormonal profile, tumor volume, and ophthalmic parameters: visual acuity (VA), perimetry, and retinal analysis using optical coherence tomography (OCT).

**Results:**

Surgery normalized ACTH, cortisol, IGF-1, and prolactin levels. Larger tumor volumes were observed in males, nonfunctioning tumors, nonoperated patients, and relapses. Larger tumors were associated with worse visual field, while VA remained stable. OCT showed thinning of total retina and inner retinal layers in patients with disease duration >5 years, nonfunctioning tumors, larger tumors, postoperative status, and relapse (p < 0.05). Macular ganglion cell layer thickness correlated inversely with IGF-1 and prolactin, and retinal nerve fiber layer with tumor size. The inner nuclear layer exhibited a non-linear behavior, thickening in early stages while thinning in severe injury.

**Conclusion:**

Hormonal and radiological factors are associated with visual dysfunction. OCT and perimetry detect early visual pathway damage better than VA. Non-linear behavior of the INL stand out as a potential prognostic biomarker.

## Introduction

The pituitary gland is a neuroendocrine organ located at the base of the skull. It is housed within the sella turcica of the sphenoid bone and is responsible for secreting multiple trophic hormones, oxytocin, and antidiuretic hormone. Its anatomical position is critical because it lies close to multiple vascular and neural structures. Notably, its anterior and lateral sides are contiguous with structures essential for normal vision (1, 2). This proximity explains why expansive pituitary tumors and/or treatment-related iatrogenic effects can lead to visual disturbances (3).

Pituitary tumors account for 10–15% of intracranial neoplasms (4). The most frequent subtype is the pituitary adenoma, comprising approximately 90% of these lesions. Its prevalence in the general population is estimated at 14–23% and is higher in females (5, 6). Although benign, pituitary adenomas can produce symptoms due to mass effect on adjacent structures and through dysregulated hormone secretion. Adenomas are classified as macroadenomas when any dimension exceeds 10 mm, and as functioning adenomas when they secrete active hormones—most commonly prolactin-secreting prolactinomas (associated with amenorrhea, galactorrhea, infertility, and sexual dysfunction), followed by somatotroph adenomas secreting GH (causing acromegaly), corticotroph adenomas secreting ACTH (causing Cushing disease), and, rarely, thyrotroph adenomas secreting TSH, and gonadotroph adenomas secreting LH or FSH (7).

Pituitary macroadenomas manifest ophthalmically by compressing the optic chiasm, optic nerves, and adjacent structures. The most characteristic clinical sign is a visual field defect. Other neuro-ophthalmic manifestations include diplopia and retro-orbital headache. Axons from nasal retinal fibers, which decussate at the optic chiasm, transmit information from the temporal visual field. Direct chiasmal compression impairs transmission of the visual signal to the occipital cortex and manifests as a visual field defect, most typically bitemporal hemianopia. Depending on tumor growth, unilateral defects may occur if one optic nerve is predominantly involved. Asymmetric defects may also be observed. When compression is severe, chronic and advanced, neuroretinal alterations can be detected by ophthalmic examination of the optic nerve head and macula. Axonal injury also propagates retrogradely, leading to retinal ganglion cell death and consequent reductions in visual acuity, impaired color vision, and ultimately, optic atrophy without potential for recovery.

OCT enables cross-sectional imaging of the retina and optic nerve in near-histological detail. As a light-based imaging technique, provides non-invasive micron-resolution images of retinal structure. It is particularly valuable for diagnosing and monitoring conditions such as glaucoma, optic neuropathies, papilledema, and optic atrophy (8–11). It also yields information about trans-synaptic retrograde degeneration in post-geniculate lesions. Its measurements are objective and reproducible using tracking and anatomical recording systems that enable precise, repeated assessment at the same retinal location.

Neuro-ophthalmic manifestations may precede or accompany the endocrine diagnosis (3). The severity of visual field defects and the degree of retinal nerve fiber layer (RNFL) thinning measured by OCT correlate with the likelihood of visual recovery after decompressive surgery. Early detection enables treatment initiation before irreversible damage occurs (12–14).

## Materials and methods

### Study design and population

A retrospective observational study was conducted on adult patients (18–80 years) diagnosed with pituitary macroadenoma and under follow-up by the Departments of Endocrinology, Neurosurgery, Radiology and Ophthalmology at Miguel Servet University Hospital, Zaragoza, between January 2020 and December 2023. This 4-year period served as the inclusion timeframe for unique individuals. No longitudinal repeated measurements were performed, except for patients who underwent surgery, who were followed over time to monitor the effects of pituitary tumor resection on visual function. All subjects had provided written informed consent. The study was approved by the Research Ethics Committee of the Autonomous Community of Aragon (PI24/082) and conducted in accordance with the principles of the Declaration of Helsinki. Patients were excluded if they presented refractive errors—high myopia (< -6 diopters), high hyperopia, or astigmatism (≥ 3 diopters)—or best-corrected visual acuity (BCVA) < 0.05. Additional exclusions were intraocular pressure (IOP) > 20 mmHg and any ocular or neurological condition that could interfere with examination results (unilateral or bilateral amblyopia; prior diagnosis of optic neuropathy: glaucoma, toxic, hereditary, or malformity etiologies). The inclusion criteria specifically required the presence of a macroadenoma with anatomical risk of visual pathway compression. A total of 40 patients were included in the study.

### Data sources

**Figure 1:**
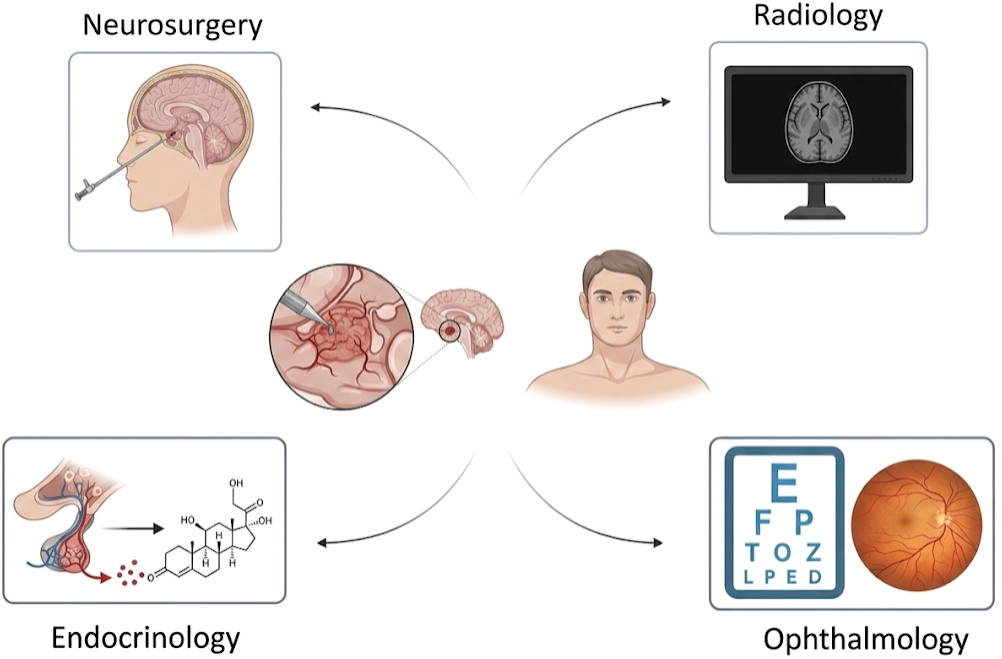
Hospital departments involved in the multidisciplinary management of patients diagnosed with pituitary tumors. Illustration created with the BioRender app.

Demographic (age, sex) and clinical variables (disease duration and medical or surgical treatment) were collected. Analytical data were retrieved from the Modulab centralized institutional repository to assess tumor hormonal activity—specifically, TSH, T4, LH, FSH, prolactin, ACTH, cortisol, GH, and IGF-1 levels. To ensure temporal synchronization, ophthalmological examinations were scheduled close to the endocrine evaluations, typically following direct referral from the endocrinology department. For patients with multiple hormonal assessments within that year, the arithmetic mean of the values was computed to account for potential temporal fluctuations. To avoid bias from sex- and age-dependent cutoffs, hormones were dichotomized as “abnormal” vs. “normal” according to laboratory reference ranges, and analyses prioritized abnormal values. Reference (normal) hormonal ranges are provided in **Supplementary Material 1**. Pituitary gland magnetic resonance imaging (MRI) data were obtained from the RIS–PACS centralized institutional repository. Tumor progression was monitored using high-resolution MRI with submillimeter slices. Using advanced image processing software (ImagenSalud), multiplanar and 3D reconstructions were generated to identify the maximum diameters in the coronal, sagittal, and axial planes. Tumor volume was subsequently calculated using the ellipsoid formula (0.524 X d1 X d2 X d3) and expressed in cubic millimeters (mm^3^). The degree of contact, compression or chiasmatic displacement and tumor invasion using the Knosp classification system, were analyzed. Relapse of tumor was considered as a group of any kind of post-surgery tumor detectable by imaging, including true recurrences and postoperative remnants. Recurrence implies regrowth after confirmed complete resection and residual tumor implies incomplete surgery. These are different clinical and prognostic entities, but the two entities were pooled due to small numbers (**Figure 1**).

### Ophthalmic examination

**Figure 2:**
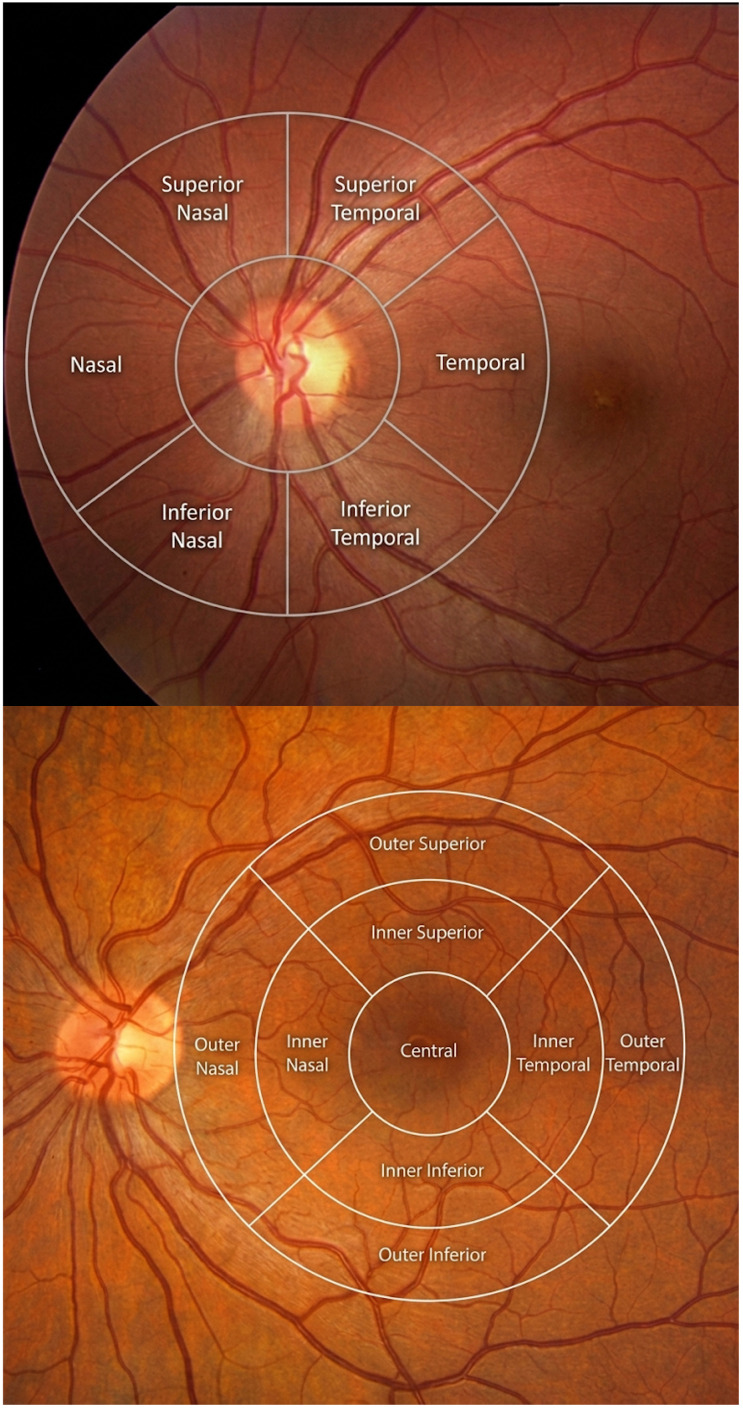
Fundus photograph (top) of the macular region showing the ETDRS (Early Treatment Diabetic Retinopathy Study) sectors and of the optic nerve head (bottom) showing the peripapillary sectors analyzed by optical coherence tomography (OCT), displayed as an overlay.

The ophthalmic parameters analyzed were BCVA, IOP, visual field (VF), and OCT. BCVA was assessed monocularly using 100%-contrast Snellen optotypes at 6 meters under mesopic illumination. IOP was measured by Goldmann applanation tonometry, recording the mean in mmHg. For VF testing, a Humphrey perimeter (Zeiss Meditec, Dublin, CA) was used with Goldmann size-III static, achromatic stimuli (duration 100–200 ms), and an achromatic background of constant luminance. The Swedish Interactive Threshold Algorithm Standard (SITA Standard) and the 30–2 program were employed. The latter evaluates the central 30° relative to the macula. The indices analyzed were visual field index (VFI), mean deviation (MD), and pattern standard deviation (PSD); reliability indices included fixation losses (FL), false positives (FP), and false negatives (FN). VF reliability criteria were FL < 25%, FP < 15%, and FN < 15%. Structural analysis of the retina and optic nerve was performed with the Spectralis^®^ OCT device (Heidelberg Engineering, Germany). High-resolution, eye-tracking protocols were applied for macular (central retina) and peripapillary (optic nerve) assessments to ensure precise, repeated measurements at identical locations throughout the study. All OCT scans were acquired by the same experienced examiners to ensure consistency. Image quality control was strictly managed using the device’s quality score, and only scans with an optimal Q Score > 25 were included in the analysis. Retinal layer segmentation was performed automatically by the built-in software of the OCT device; however, all automated segmentations were systematically reviewed by the investigators using the Thickness Map tool to identify and rule out any potential segmentation artifacts. These protocols quantified the thickness (µm), by sector, of the total retina and, after segmentation, of the retinal nerve fiber layer (RNFL; peripapillary and macular), ganglion cell layer (GCL), inner plexiform layer (IPL), and inner nuclear layer (INL), outer plexiform layer (OPL), outer nuclear layer (ONL), retinal pigment epithelium (RPE). For the evaluation of retrograde neurodegeneration, only the inner layers of the retina were analyzed. At the macula, sectors followed the 9-zone ETDRS grid (central; inner and outer rings subdivided into superior, inferior, nasal, and temporal) (**Figure 2**, top). At the peripapillary level, the sectors analyzed were nasal, superior, and inferior; and temporal, superior, and inferior (**Figure 2**, bottom). OCT parameters were evaluated using the Spectralis normative database for age, sex and ethnicity. Pathological peripapillary neuroretinal thinning was defined as a thickness measurement falling within the <1% distribution (marked as ‘outside normal limits’). For macular thickness analysis, a longitudinal approach was employed, defining thinning as a significant decrease in thickness relative to the patient’s initial baseline scan, ensuring that each patient served as their own internal control for progression analysis. Participants underwent annual OCT imaging. To ensure data consistency, when multiple scans were available within the same calendar year, the arithmetic mean of the retinal layer thickness measurements was calculated and utilized for analysis. This longitudinal approach provided both preoperative and postoperative structural data for all patients who underwent surgical intervention, enabling a comparative assessment of neuroretinal changes over time.

### Statistical analysis

This study was conducted using retrospectively processed, pseudonymized data compiled as a database in Microsoft^®^ Excel. When multiple examinations were available for the same patient at a given time point, the values were averaged to yield a single value per patient per year. Statistical analyses were performed using IBM SPSS Statistics, version 20.0. Kolmogorov-Smirnov test showed that most of the variables followed a normal distribution; for this reason, parametric tests were performed. A descriptive statistics and comparative analyses were performed using Student’s *t*-test and ANOVA. To avoid statistical bias associated with inter-ocular dependency, only one eye per patient was randomly selected for data analysis. Evaluated variables included age, sex, and BCVA; VF indices (VFI, MD, PSD) and reliability metrics (FP, FN, FL); sectoral retinal thickness at the macular and peripapillary regions; tumor duration (< 5 vs. ≥ 5 years), tumor functionality, hormonal abnormality, tumor size, anatomical variables related to contact, compression, chiasmatic displacement, and invasion (Knosp grade), surgical treatment, and occurrence of relapse. The rationale for the 5-year cutoff regarding tumor duration was a data-driven decision based on our pituitary tumor cohort and aimed at obtaining groups balanced in terms of sample size. Pearson’s correlation was used to assess associations. Statistical significance was set at p < 0.05, with Bonferroni correction applied for multiple comparisons by dividing the significance level of 0.05 by the number of comparisons shown in each table. In other words, if the table shows 10 comparisons, the significance level is reduced to 0.005.

## Results

Of the 141 patients identified, 40 met inclusion criteria and were analyzed. Mean age was 59.2 ± 12.3 years, with females predominating (60%). Over the 4-year observation period, 28 of the 40 patients were incident cases (average of 7 new cases per year), and 12 were prevalent cases. All tumors were macroadenomas (largest dimension > 10 mm) and mean disease duration was 8.08 ± 7.33 years. Based on pituitary hormonal secretion, 85% of the tumors were nonfunctioning. Nonetheless, hormonal abnormalities were detected in 13 out of 40 of patients (32.5%), with prolactin being the most frequently monosecreted hormone altered (12,5%). The rest of the cohort with hormonal alterations showed a co-secretion profile, with ACTH being the most frequently altered (17.5%), followed by TSH and LH (both 12.5%), FSH (10%). FSH levels remained below the normal range throughout the entire study period (see **Supplementary Material 2**). Surgery was performed on 67.5% of patients, of whom 37.5% presented tumor relapse during follow-up, and 25% also received radiotherapy; additionally, 22.5% were treated with cabergoline. On ophthalmic evaluation, BCVA was good and remained stable throughout the study period, and intraocular pressure remained within the normotensive range (< 21 mmHg). Greatest functional deterioration was observed in 2021, when visual field indices and mean deviation presented the worst values. Total retinal thickness remained within normal limits; however, the RNFL, GCL, and IPL showed thinning (**Table 1**).

**Table 1:** Ophthalmic descriptive data in patients with pituitary macroadenoma.

YEAR	2020	2021	2022	2023
Visual acuity				
	0.80 ± 0.15	0.76 ± 0.25	0.70 ± 0.31	0.79 ± 0.27
Intraocular pressure (mmHg)				
	14.83 ± 3.92	16.65 ± 2.15	15.44 ± 2.62	15.47 ± 1.68
Visual field				
FP (%)	1.80 ± 2.02	1.92 ± 2.56	2.58 ± 4.09	2.95 ± 3.33
FN (%)	6.26 ± 7.18	5.21 ± 7.18	9.72 ± 14.53	7.08 ± 10.43
FL	13.32 ± 19.88	8.02 ± 8.99	7.91 ± 12.80	9.63 ± 13.60
VFI (%)	89.68 ± 11.30	80.52 ± 18.11	88.33 ± 15.88	92.61 ± 10.70
MD (dB)	-4.33 ± 4.29	-10.11 ± 32.17	-4.98 ± 5.70	-3.52 ± 4.09
PSD (dB)	4.92 ± 3.75	8.51 ± 5.64	5.51 ± 4.16	4.53 ± 3.98
Optical coherence tomography (μm)				
RETINA	273.32 ± 84.93	310.96 ± 29.17	297.42 ± 31.67	301.8 ± 21.75
RNFL	24.48 ± 5.70	27.09 ± 15.64	24.84 ± 9.16	24.79 ± 9.09
GCL	33.95 ± 6.93	33.94 ± 9.94	32.01 ± 7.60	33.91 ± 6.35
IPL	30.40 ± 5.35	30.04 ± 8.37	29.20 ± 5.89	30.52 ± 4.61
INL	35.33 ± 5.01	36.36 ± 5.24	35.58 ± 5.79	35.43 ± 4.38

VFI, Visual field index; MD, mean deviation; PSD, pattern standard deviation; RNFL, retinal nerve fiber layer; GCL, ganglion cell layer; IPL, inner plexiform layer; INL, inner nuclear layer.

Accepted visual field reliability thresholds: false positives (FP) < 15%, false negatives (FN) < 15%, fixation losses (FL) < 25%.

### Analysis of hormonal changes

**Figure 3:**
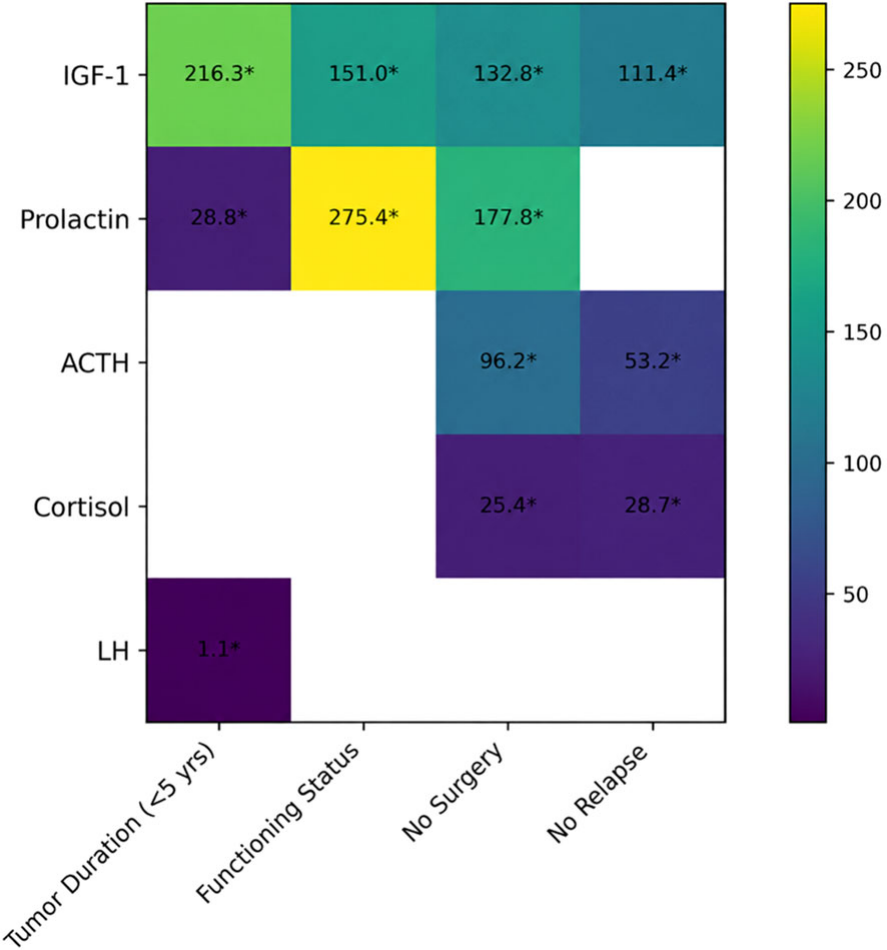
Integrative heatmap of hormonal profiles and clinical tumor characteristics. This matrix illustrates the statistically significant associations (* = p<0.05) between altered hormonal levels and specific clinical features. Color intensity represents the mean hormonal concentration, highlighting the prominence of IGF-1 and Prolactin across multiple clinical states, and the association of ACTH and Cortisol with non-surgical and non-relapse status. insulin-like growth factor 1 (IGF-1), adrenocorticotropic hormone (ACTH), luteinizing hormone (LH).

Hormonal changes were analyzed according to tumor duration, functional status, surgery, and relapse (**Figure 3**). Statistically significant differences (p < 0.05) were found in 5 hormones: prolactin, ACTH, cortisol, LH, and IGF-1. Tumors with a duration < 5 years showed higher levels of LH (1.05 ± 0.01 vs. 0.24 ± 0.05 mIU/mL; n= 1 vs. 2), IGF-1 (216.30 ± 218.37 vs. 56.56 ± 11.70 ng/mL; n= 3 vs. 5), and prolactin (28.83 ± 45.35 vs. 6.15 ± 9.89 ng/mL; n= 11 vs. 10) (p < 0.05). Functioning tumors secreted higher levels of IGF-1 (133.40 ± 112.79 vs. 60.97 ± 33.77 ng/mL; n= 3 vs. 8) and prolactin (275.38 ± 407.33 vs. 13.84 ± 33.00 ng/mL; n= 5 vs. 13) than nonfunctioning tumors (p < 0.05). Patients who did not undergo surgery had higher levels of ACTH (96.20 ± 0.01 vs. 26.57 ± 25.17 pg/mL; n= 2 vs. 1), cortisol (25.43 ± 0.01 vs. 6.34 ± 4.74 µg/dL; n= 1 vs. 10), IGF-1 (144.46 ± 105.48 vs. 56.82 ± 31.04 ng/mL; n=3 vs. 8), and prolactin (35.15 ± 46.79 vs. 6.25 ± 7.82 ng/mL; n= 7 vs. 14) (p < 0.05), whereas postoperative patients exhibited hormonal normalization. Patients without relapsing tumors had higher ACTH (53.23 ± 2.14 vs. 25.70 ± 0.01 pg/mL; n= 3 vs. 1), cortisol (28.74 ± 11.17 vs. 2.90 ± 0.97 µg/dL; n= 2 vs. 4), and IGF-1 (111.36 ± 36.98 vs. 67.47 ± 15.96 ng/mL; n= 3 vs. 4) levels than those with relapse; notably, relapsing cases showed subnormal cortisol levels (2.90 ± 0.97 vs. 28.74 ± 11.17 µg/dL) (p < 0.05). It should be noted that all subgroups, except for those related to prolactin, had a sample size of less than 5 in at least one subgroup; therefore, in these cases, the data should be considered descriptive.

### Radiological analysis

**Figure 4:**
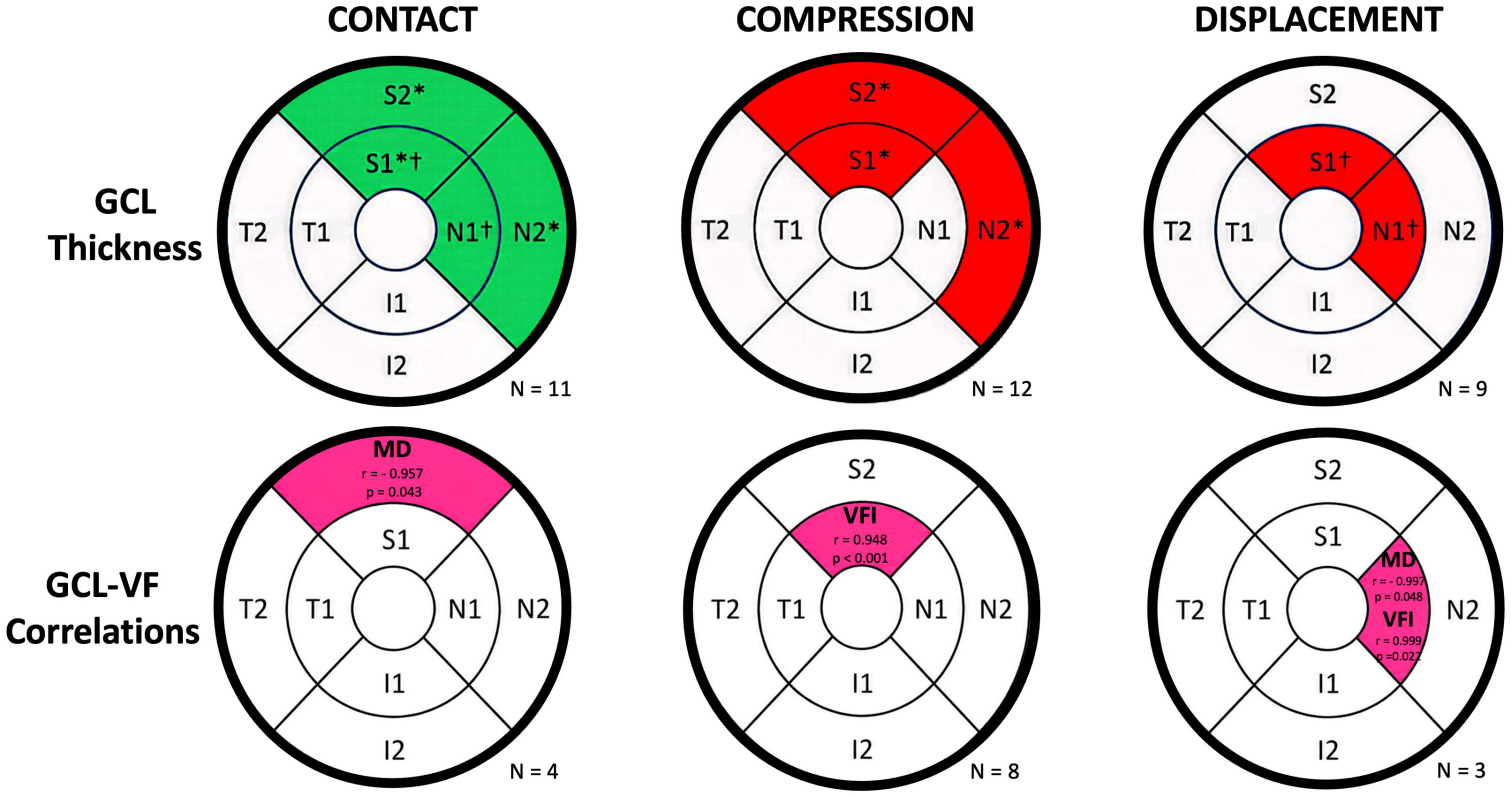
Ganglion cell layer (GCL) thickness and visual field (VF) correlations according to tumor-chiasm anatomical variables. Macular sectors: S, superior; I, inferior; N, nasal; T, temporal; I, inner; 2, outer; VFI, visual field index; MD, mean deviation. Colored sector if statistical differences (ANOVA: p < 0.05); green, normal thickness; red, reduced thickness; pink, structure-function correlation, *statistical differences between contact and compression; †statistical differences between contact and displacement; r, Pearson correlation coefficient; N; sample number.

One-third (32.4%) of patients exhibited chiasmatic contact, 35.3% compression, and 32.4% also displacement. Most patients (40%) had cavernous sinus invasion grade I on the Knosp scale, 20% grade II, 10% grades 0 and IV, respectively, and 6.7% grade III. When evaluating the impact of these anatomical relationships on structural and functional outcomes, a comparative analysis revealed statistically significant differences in GCL thickness among the tumor anatomical variables. Specifically, the chiasmatic contact group consistently maintained a significantly greater GCL thickness compared to the compression and displacement groups. Furthermore, subsequent correlation analysis identified significant associations between these anatomical stages and specific visual field sectors and parameters, despite the limitation of a small sample size in some subgroups. In contrast, no statistically significant differences or trends were observed across the different grades of the Knosp scale regarding either OCT or visual field metrics (**Figure 4**). Radiological (MRI) data demonstrated statistically significant differences in tumor size. Larger tumors were identified in males than in females (32,315.66 ± 28,704.88 vs. 9,792.00 ± 10,521.84 mm³ in 2021, and 10,273.50 ± 7,489.91 vs. 3,459.85 ± 1,814.87 mm³ in 2023; p < 0.05), in patients with nonfunctioning tumors (5,698.50 ± 6,134.49 vs. 1,134.25 ± 8,021.25 mm³; p < 0.05), in patients without surgery (22,044.57 ± 22,294.02 vs. 5,242.75 ± 3,698.57 mm³; p < 0.05), and in cases of relapse (26,646.20 ± 24,759.67 vs. 7,008.66 ± 5,826.31 mm³; p < 0.05). And of the total number of 40 patients evaluated, 17 experienced tumor relapses during the study period (42.5%), considered a recurrence or a postoperative remnant.

### Ophthalmic analysis

Ophthalmic testing revealed changes in functional and structural values. Patients with pituitary macroadenomas had better visual acuity when tumor duration exceeded 5 years (0.90 ± 0.14 vs. 0.64 ± 0.28; p < 0.05) and when tumor size was < 10,000 mm³ (0.88 ± 0.18 vs. 0.69 ± 0.31; p < 0.05). Although IOP remained within normotensive levels (IOP < 21 mmHg), it was lower in patients who had relapsing tumors (13.95 ± 2.09 vs. 16.61 ± 2.42 mmHg; p < 0.05).

The worst perimetric values were recorded in patients with larger tumors (> 10,000 mm³), who showed higher false-negative rates (11.00 ± 9.20 vs. 2.85 ± 4.20%; p < 0.05), lower VFI (11.00 ± 3.85 vs. 85.90 ± 15.58%; p < 0.05), greater negative MD (−5.02 ± 3.78 vs. −2.04 ± 3.07 dB; p < 0.05), and higher PSD (12.76 ± 3.85 vs. 7.23 ± 5.42 dB; p < 0.05). Patients with disease duration > 5 years had more fixation losses (0.13 ± 0.17 vs. 0.04 ± 0.06%; p < 0.05) and false negatives (10.50 ± 8.52 vs. 3.10 ± 5.56%; p < 0.05). Those who underwent surgery (−6.89 ± 5.91 vs. −3,020.31 ± 5.23,46 dB; p < 0.05) or had nonfunctioning tumors (−5.91 ± 6.12 vs. −4.22 ± 5.89 dB; p < 0.05) exhibited lower MD. No statistically significant differences were found with respect to relapse.

**Figure 5:**
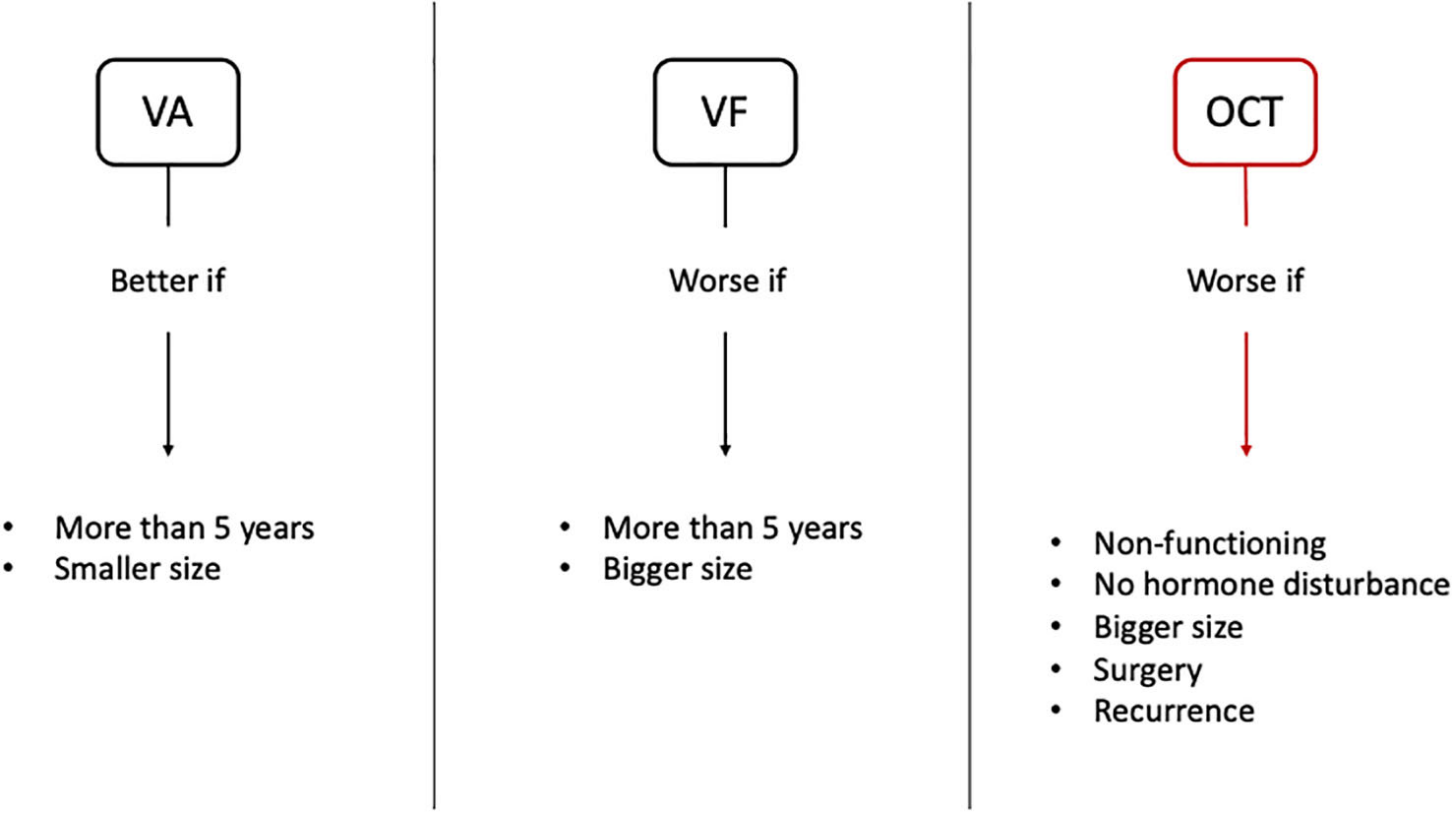
Ophthalmic examination results and their relationship with the tumor parameters studied. VA, visual acuity; VF, visual field; OCT, optical coherence tomography.

**Figure 6:**
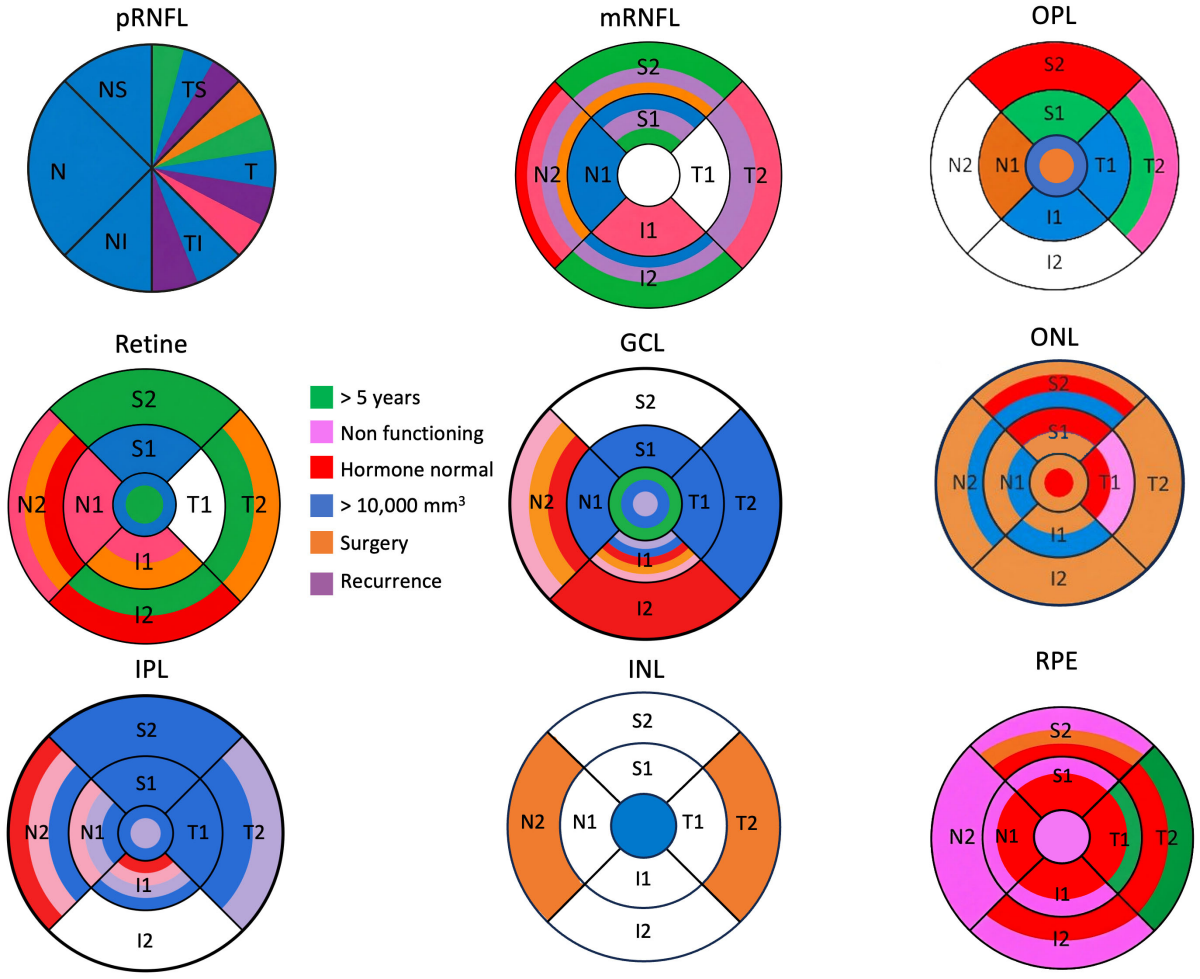
Structural neuroretinal changes detected by optical coherence tomography (OCT) in patients with pituitary macroadenomas. Macular and peripapillary sectors are color-coded where thickness decreased (p < 0.05). GCL, ganglion cell layer; IPL, inner plexiform layer; INL, inner nuclear layer; mRNFL, macular retinal nerve fiber layer; pRNFL, peripapillary retinal nerve fiber layer; OPL, outer plexiform layer; ONL, outer nuclear layer; RPE, retinal pigment epithelium. Macular sectors: S1, superior inner; S2, superior outer; I1, inferior inner; I2, inferior outer; N1, nasal inner; N2, nasal outer; T1, temporal inner; T2, temporal outer. Peripapillary sectors: NS, nasal–superior; N, nasal; NI, nasal–inferior; TI, temporal–inferior; T, temporal; TS, temporal–superior.

As illustrated in **Figure 5**, OCT detected changes across a broader set of tumor characteristics. Structural OCT analysis showed both reduced total neuroretinal thickness and thinning of inner retinal layers—pRNFL, mRNFL, GCL, and IPL—in patients with tumor follow-up > 5 years, in patients with hormonally nonfunctioning tumors, in patients with normalized hormone levels, in patients with larger tumors (> 10,000 mm³), after surgery, and in patients with relapsing tumors (p < 0.05). Within the inner layers, the INL decreased solely in patients with large tumors or after surgery (p < 0.05); in all other scenarios, INL thickness increased significantly (see **Figure 6** and **Supplementary Material 3**).

**Figure 7:**
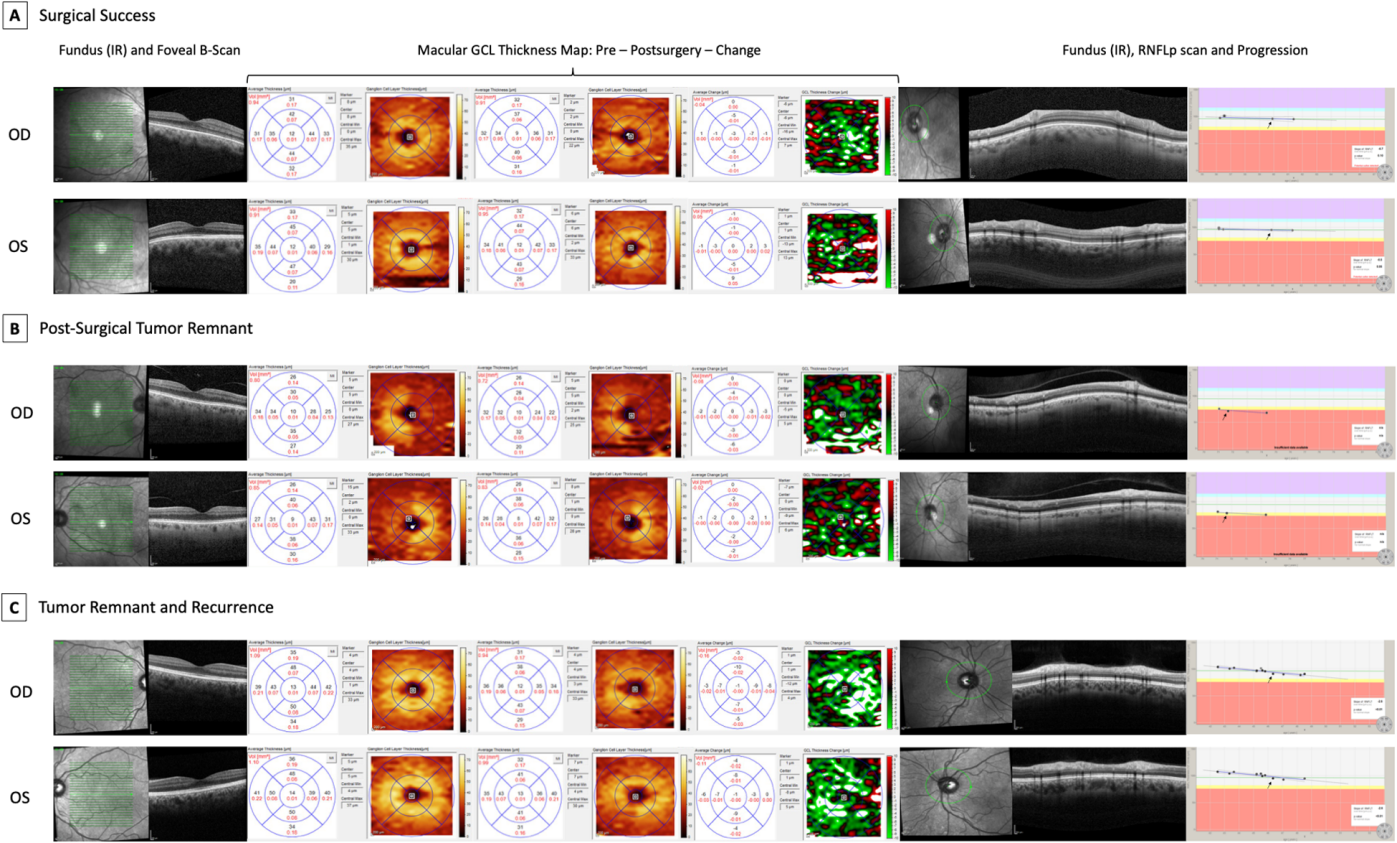
Optical coherence tomography progression analysis of macular ganglion cell layer (GCL) and peripapillary retinal nerve fiber layer (RNFLp) thickness across three clinical scenarios. The panels illustrate a gradient of prognostic outcomes: **(A)** optimal neuroretinal preservation after complete tumor removal, **(B)** moderate progressive thinning in case of tumor remnant, and **(C)** progressive structural loss in the worst-case scenario, tumor remnant and recurrence in which significant thinning is observed (in green). The arrow indicates the preoperative scan. GLC, Ganglion cell layer; IR, Infrared; OD, Right eye; OS, Left eye; µm, micrometer.

**Figure 8:**
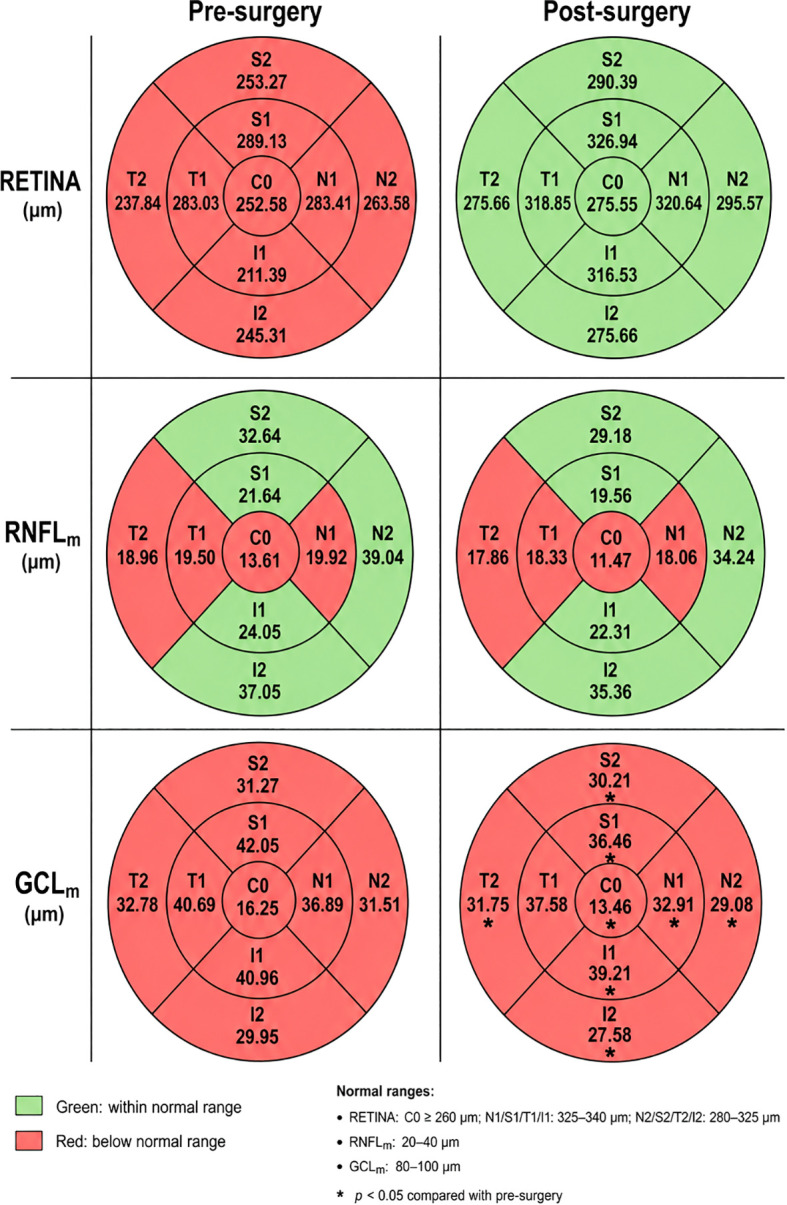
Pre- and postoperative structural changes in retinal thickness by layer and sector in patients with pituitary tumors. S, superior; T, temporal; I, inferior; N, nasal; 1, inner. 2, outer; RNFL, retina nerve fiber layer; GCL, ganglion cell layer.

A longitudinal analysis was conducted to compare preoperative baseline measurements (2020) with post-surgical outcomes (2023) for the 27 patients who underwent tumor resection (**Figure 7**). By the 2023 assessment, all surgical patients had a minimum follow-up of one-year post-intervention. Tumor size decreased from 13,453.46 ± 8,985.66 mm³ to 6,726.4 ± 1,305.419 mm³ after surgery and was smaller than in those who did not undergo surgery (5,475.00 ± 6,253.762 mm^3^) (p = 0.001). As detailed in **Table 2**, surgical intervention led to a significant improvement in functional visual parameters, characterized by an increase in the Visual Field Index (VFI) and a reduction in Mean Deviation (MD) and Pattern Standard Deviation (PSD) deficits. In contrast, a significant reduction in neuroretinal structural integrity was observed postoperatively. This structural loss was particularly pronounced and widespread within the ganglion cell layer thickness (**Figure 8**, **Supplementary Material 4**), highlighting a divergence between functional recovery and progressive neuroretinal thinning. Among the 27 patients who underwent surgery, only 8 patients (29.6%) achieved complete tumor resection with no evidence of recurrence during follow-up, 9 had radiologically detectable postoperative remnants, and 10 developed either tumor recurrence or persistent remnant disease during follow-up. Those who experienced relapse showed a significant change in the hormones TSH, T4, LH, prolactin, cortisol, and the visual field parameters VFI, DM and PSD (p<0.05).

**Table 2:** Pre- and postoperative functional and structural visual outcomes in patients with pituitary tumors.

Ophthalmic parameters	Pre-surgery	Post-surgery	p
BCVA	0.87 ± 0.15	0.80 ± 0.23	0.808
IOP	16.50 ± 0.70	10.00 ± 0.01	0.598
Fixation losses VF	0.11 ± 0.22	0.06 ± 0.06	0.466
False positive VF	1.65 ± 1.87	2.50 ± 2.85	**0.004**
False negative VF	5.34 ± 3.89	6.07 ± 9.20	0.472
VFI VF	86.84 ± 13.74	90.46 ± 12.06	**0.043**
MD VF	-5.184 ± 4.73	-4.14 ± 4.88	**0.012**
PSD VF	5.82 ± 4.36	4.63 ± 4.58	**0.002**
RETINA Total Volume mm3	8.46 ± 0.57	8.26 ± 0.56	**<0.001**
RNFL Center µm	6.66 ± 18.71	3.68 ± 5.32	**0.033**
GCL Total Volume mm3	0.93 ± 0.13	0.86 ± 0.09	**<0.001**

BCVA, best corrected visual acuity; IOP, intraocular pressure; VF, visual field; VFI, visual field index; MD, mean deviation. PSD, pattern standard deviation; RNFL, retina nerve fiber layer; GCL, ganglion cell layer. Bold values: p<0.05.

### Correlation analysis

BCVA correlated inversely with cortisol, prolactin, and IGF-1 (r = −0.795; p = 0.006), and directly with OCT-measured thicknesses of the total retina (sector T1), mRNFL (C0), and GCL (C0: r = 0.727; p = 0.005; T1: r = 0.763; p = 0.001; I1: r = 0.978; p = 0.022); However, BCVA correlated inversely with GLC thickness in sector S1. No strong correlation was found between tumor size and BCVA. Tumor size correlated inversely with mRNFL in sectors N1, N2, S2, and I2 (r = −0.829; p < 0.005).

**Figure 9:**
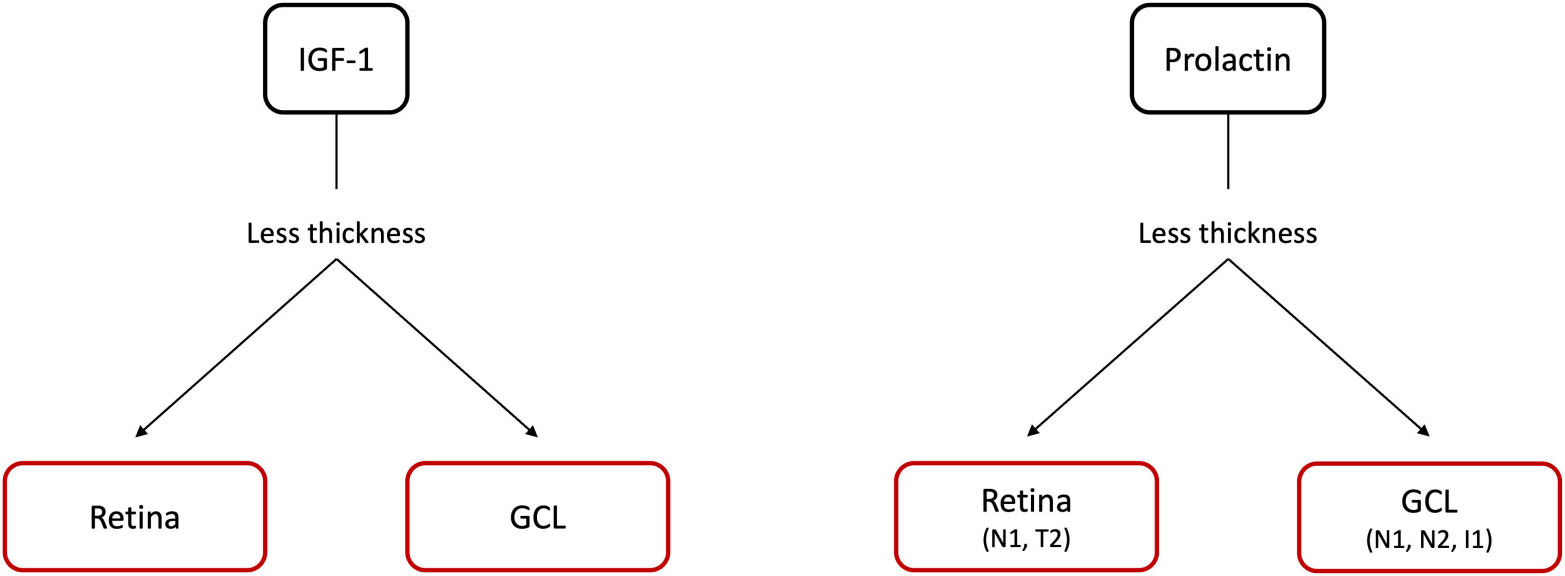
Strong correlations identified between abnormal hormone levels and reduced neuroretinal thickness detected by optical coherence tomography (OCT). insulin-like growth factor-1 (IGF-1), ganglion cell layer (GCL), nasal (N), temporal (T), inferior (I), inner (1), outer (2).

A strong inverse correlation was observed between neuroretinal thickness and several hormones: IGF-1 correlated with total retinal thickness (r = −0.838; p = 0.002) and with GCL across all sectors (r = −0.804; p < 0.005); prolactin correlated with total retinal thickness in sectors N1 and T2, and with GCL in N1 (r = −0.863; p < 0.005), N2 (r = −0.819; p = 0.001), and I1 (r = −0.820; p = 0.001). This strong inverse correlation was maintained in a stratified subanalysis after excluding those patients treated with cabergoline, which directly suppresses prolactin secretion. GCL in N1 (r = −0.852; p=0.007); N2 (r = −0.873; p = 0.005). These findings reinforce this correlation. The visual field showed no strong correlations with any of the analyzed parameters (**Figure 9**).

To further evaluate the relationship between structural retinal parameters and tumor burden, a multivariate logistic regression analysis was performed using baseline measurements. Tumor size was treated as the dependent variable, dichotomized into two groups: <10.000 mm^3^ and ≥10.000 mm^3^. The model included total retinal volume, mean retinal thickness, retinal nerve fiber layer thickness, and ganglion cell layer thickness as predictor variables. The multivariate analysis confirmed that all four structural parameters were significant independent predictors of tumor volume (p<0.05 for all variables). These findings suggest that baseline neuroretinal thinning and volume reduction are closely associated with larger tumor dimensions at the time of diagnosis.

## Discussion

This study examines patients with pituitary macroadenomas to identify which hormonal parameters (functionality and hormonal dysregulation), clinical variables (sex, surgery, relapse), and tumor features (size and duration) are associated with greatest deterioration of visual function and neuroretinal structure, and which ophthalmic parameters show the most pronounced changes. By integrating these disparate factors, we emphasize the necessity of a multidisciplinary approach, where endocrine, radiological, and ophthalmological data are not viewed as isolated parameters but as an interconnected clinical reality.

From an endocrine perspective, we investigated whether hormonal abnormalities could influence visual impairment independently of tumor size. Although optic chiasm compression remains the main determinant of visual dysfunction, the endocrine profile may reflect tumor biological activity and factors affecting neuronal resilience. IGF-1, prolactin, and ACTH were the most frequently altered hormones. Higher IGF-1 and prolactin levels in functioning tumors of shorter duration (<5 years) likely reflect earlier clinical detection due to hormonal hypersecretion rather than compressive symptoms alone. Previous studies have reported conflicting results regarding the relationship between hormonal abnormalities and retinal structure, with some attributing retinal changes exclusively to tumor compression and others highlighting the importance of controlling GH/IGF-1 excess to reduce tumor burden and improve surgical outcomes (15, 16). We observed inverse correlations between prolactin levels and retinal thickness, particularly at the GCL level, could suggest a deleterious role of prolactin in the retina, although prolactin has also been proposed to exert neuroprotective effects under certain conditions (17). Higher ACTH, cortisol, and IGF-1 levels among non-operated patients may reflect clinical selection toward medically manageable phenotypes (18). Finally, the inverse correlation between visual acuity and IGF-1, cortisol, and prolactin levels could suggest a potential link between hormonal activity, tumor biology, and visual function. While IGF-1 and prolactin have been implicated in retinal angiogenic pathways, the role of the prolactin/vasoinhibin axis in retinal homeostasis remains incompletely understood (19–22).

Imaging revealed that the largest tumor volumes were found in males, in nonfunctioning tumors, in patients without surgery, and in those with relapse. The greater volume in males aligns with prior papers and may reflect, in addition to potential biological differences, delayed diagnosis due to less conspicuous symptoms (23–25). The association between lack of surgery and larger volume is expected based on indication, and relapse inherently entails a significant post-surgery tumor presence. Larger tumors were associated with worse perimetric values and greater thinning of inner retinal layers. Postoperative hormonal normalization reinforces the value of surgery, not only to alleviate mechanical compression of adjacent structures but also to restore glandular secretory function.

Ophthalmologically, while visual acuity tended to remain unchanged, earlier abnormalities were revealed on visual field testing and, most notably, on OCT-based structural retinal assessment. Visual acuity was better in tumors with > 5 years’ duration and with sizes > 10,000 mm³, while visual field performance worsened under these conditions. The association between longer tumor duration and better visual acuity may reflect survivorship or referral bias. Patients with prolonged follow-up in tertiary care settings are more likely to have indolent tumors with limited visual impact, whereas visual deterioration frequently triggers earlier medical or surgical intervention, reducing the observed duration of tumor follow-up. Consequently, longer tumor duration may primarily identify patients with more stable disease and preserved visual function rather than a protective effect of time itself. The poorer perimetric value (more negative MD) observed in patients who had received surgery and in those with nonfunctioning tumors could be associated with a combination of surgical impact and irreversible baseline damage, as these tumors were larger and may have left residual axonal sequelae. Previous studies identified tumor size as the most important predictor of future visual dysfunction; others have qualitatively reported that, depending on tumor size and the relative position of the tumor and optic chiasm, tumor size influences both the severity of field defects and the variety of patterns (26, 27). Our study yielded consistent findings: greater compressive severity was significantly associated with a thinner ganglion cell layer and an increased number of impaired visual field parameters, showing a strong correlation between these metrics.

The divergent trajectories of visual acuity and structural/functional damage are characteristic of compressive optic neuropathy, in which axons and ganglion cells in the nasal hemiretina are affected first, while central vision is compromised later (28). We found no correlation between tumor size and visual acuity, but there was an inverse correlation between tumor size and RNFL and GCL thicknesses in the macular region. This discordance underscores that structural damage can often be detected before manifesting functional impairment, positioning OCT as a valuable complementary biomarker for early diagnosis. As an objective modality, OCT detected change across a greater number of tumor characteristics than subjective measures (visual acuity and perimetry). In line with prior work, functional visual field changes tend to follow structural loss because 30–50% GCL loss may be required before functional defects become apparent; accordingly, up to 30% of patients with macroadenoma may show no visual field abnormalities (29). This structural-functional mismatch is clinically insightful, as it highlights a window of subclinical axonal damage where visual acuity and fields remain preserved, yet the optic pathway is already compromised. Notably, functional deterioration clustered in 2021; while this cannot be formally tested within the current study design, this pattern is temporally consistent with reported pandemic-related care delays and warrants prospective investigation. This is underscored by prior studies showing that early diagnosis is consistently associated with better postoperative recovery (30).

The association between greater tumor volume and neuroretinal thinning could possibly suggest the existence of a trans-synaptic retrograde degeneration mechanism that, although it originates on the visual pathway at the optic chiasm, affects retinal structures. Reductions in pRNFL and macular inner layers (RNFL, GCL, IPL)—observed with longer follow-up, in nonfunctioning tumors, in the absence of hormonal abnormalities, in large tumors, after surgery, and with relapse—position OCT as a sensitive biomarker of compressive damage and its trajectory. Our findings align with previous papers indicating that by identifying pRNFL thinning OCT enables early detection of structural changes in pituitary adenomas (31). Extending this concept, since we observed macular GCL alterations that correlated with visual acuity and function, we consider the macular GCL to be a better early biomarker of chiasmal compressive damage than the pRNFL, confirming the findings in prior literature (32). Moreover, macular analysis correlated more strongly with hormonal profile, tumor size, and vision parameters, and exhibited specific patterns relative to optic nerve analyses. Unlike the well-documented involvement of the RNFL and GCL, we observed a non-linear behavior in the INL, which may serve as an exploratory biomarker of disease phase and severity. INL thinning occurred only in cases of severe injury—i.e., in the context of large tumors or surgery. Conversely, INL thickening might be interpreted as an early inflammatory response or glial reactivity characterized by edema and microcystic degeneration (33). INL thickening has been documented in the context of progressive neurodegeneration and is inversely proportional to ganglion cell layer loss, suggesting a response driven by early inflammatory mechanisms, glial remodeling, or altered mechanical traction. On the other hand, the literature also accounts for instances of INL thinning, which could correspond to a chronic, late phase of structural resolution, secondary transsynaptic dropout of bipolar or horizontal cells, and ultimate tissue atrophy (34, 35). However, future histopathological studies are required to definitively confirm these underlying cellular mechanisms (36).

The inner layers underwent most changes, although significant changes were also detected in the outer layers. The OPL thinned significantly in multiple cases, so it would not be a discriminating layer. In the case of the ONL, surgery preserved its thickness significantly, and no difference was found whether the tumors had a longer duration or in cases of relapse. Furthermore, the RPE also showed no differences based on the presence or absence of relapse or tumor size. The lack of robust associations in the outer retinal layers reinforces the premise that retrograde transsynaptic degeneration predominantly impacts the inner retinal architecture. Consequently, the alterations observed in the outer layers should not be interpreted as a direct compressive effect of the tumor on the retina. Because a direct causal link between chiasmal compression and RPE variations appears unlikely, alternative mechanisms must be considered, including systemic or vascular factors, treatment-related effects, or subclinical endocrine fluctuations.

Analysis of the post-surgical impact on the visual system revealed a notable structure-function discordance. To understand this, it is worth noting that surgical patients had significantly larger tumors at baseline. Consequently, the more pronounced postoperative neuroretinal thinning should not be attributed solely to the intervention. To date, scientific evidence has not fully resolved this paradox, a phenomenon also observed in other optic neuropathies—such as glaucomatous and ischemic neuropathy—and even following stroke. In these cases, despite the resolution of the primary insult, a persistent reduction in neuroretinal thickness or accelerated ipsilateral cerebral aging is frequently reported. Several hypotheses may explain this discrepancy. From a neurophysiological perspective, cerebral plasticity may allow other cortical areas to adapt and compensate for structural loss (37), a process potentially enhanced by neuroprotective interventions such as citicoline (38). Furthermore, regarding chiasmatic decompression specifically, the recovery of axoplasmic flow may facilitate communication through preserved post-thalamic sensory-associative visual pathways (39, 40). Nevertheless, further longitudinal studies are required to corroborate these mechanisms. From a clinical standpoint, practitioners should interpret this long-term neuroretinal thinning not as active progression, but as a manifestation of delayed retrograde trans-synaptic degeneration resulting from the prior chiasmatic compression. This persistent thinning serves as a permanent morphologic marker of the patient’s history of pituitary pathology, which will likely persist even when functional visual recovery is achieved.

Future studies could benefit from incorporating advanced molecular imaging biomarkers to complement the structural and functional metrics evaluated here. Specifically, technologies such as Detection of Apoptosing Retinal Cells (DARC), which has successfully transitioned from basic laboratory science to Phase 2 clinical trials and has already been evaluated in disorders such as glaucoma and age-related macular degeneration (41), utilize fluorescently-labeled Annexin-V to allow for the non-invasive, *in vivo* visualization of cellular stress and RGC apoptosis, potentially predicting neurodegenerative progression before permanent structural thinning becomes visible on OCT. Interestingly, recent evidence indicates that *in vivo* Annexin-V labeling exhibits a complex, biphasic pattern; while it initially targets apoptosing neurons, it subsequently binds to specific subpopulations of resident microglia and infiltrating myeloid cells (42). Consequently, when available, incorporating biomarkers such as Annexin-V-based imaging in future studies could provide, dual-purpose insights: simultaneously evaluating active RGC apoptosis and the temporal course of neuroinflammatory cell infiltration during the progression of compressive optic neuropathy. Furthermore, an analysis by secretion subtype would be interesting as a future direction of research.

This study’s chief limitations are its small sample size, therapeutic heterogeneity, and the lack of internal controls. In this regard, experimental studies have reported retinal thinning associated with ageing (43, 44). However, the three-year follow-up period in the longitudinally analysed patients—who were exclusively those who had undergone surgery—could account for an additional loss of approximately 1.5 micrometres. However, the pre- and post-surgical structural change exceeded this range. Additionally, the lack of a matched non-operated control group represents a limitation, as the specific contribution of the surgical intervention to the observed structural loss cannot be isolated from the natural course of the tumor’s compressive effects. Moreover, given the observational design of this study, causal inferences cannot be definitively drawn, and further prospective studies are needed to establish the directionality of these associations. The logistic regression model should be considered exploratory given the sample size, and the radiation exposure in some patients as a potential confounder. The data-driven choice, regarding 5-year disease duration cutoff could have associated optimization risk. Generalizability is limited to pituitary macroadenomas and cannot be extrapolated to microadenomas, as all patients in this series had the former. Future research should include prospective studies; deeper characterization of the INL to track putative inflammatory structural changes and their postoperative development as a potential prognostic severity marker; and the development of predictive models that integrate tumor volume, hormonal profile, and structural/functional ophthalmic tests (OCT and perimetry).

Finally, this paper underscores that multidisciplinary collaboration is essential to achieve accurate diagnosis, timely intervention, and optimized treatment outcomes (45). Altogether, the findings highlight the suitability of comprehensive neuro-ophthalmic examination and the promising diagnostic and prognostic value of OCT for detecting subtle structural abnormalities associated with pituitary tumors (46).

## Data Availability

The raw data supporting the conclusions of this article will be made available by the authors, without undue reservation.

## Supplementary material

Supplementary Material 2 Hormone values in patients with a pituitary tumor. The normal value range is shaded in green. TSH, Thyroid-stimulating hormone. T4, Tetraiodothyronine/Thyroxine. LH, Luteinizing Hormone. FSH, Follicle-stimulating hormone. ACTH, Adrenocorticotropic hormone. GH, Growth hormone. IGF1, Insulin-like growth factor 1.

Supplementary Material 3 for Figure 6 Structural neuroretinal changes detected by optical coherence tomography (OCT) in patients with pituitary macroadenoma. Macular and peripapillary sectors are color-coded where thickness decreased (p < 0.05). Green, tumor duration > 5 years Pink, nonfunctioning tumor; Red, normal hormone levels; Blue, tumor > 10,000 mm³; Orange, surgery; Purple, relapse. GCL, ganglion cell layer; IPL, inner plexiform layer; INL, inner nuclear layer; RNFL, retinal nerve fiber layer; OPL, outer plexiform layer; ONL, outer nuclear layer; RPE, retinal pigment epithelium. m, macular sectors: S1, superior inner; S2, superior outer; I1, inferior inner; I2, inferior outer; N1, nasal inner; N2, nasal outer; T1, temporal inner; T2, temporal outer. p, peripapillary sectors: NS, nasal–superior; N, nasal; NI, nasal–inferior; TI, temporal–inferior; T, temporal; TS, temporal–superior.

Supplementary Material 4 for Figure 8 S, superior; T, temporal; I, inferior; N, nasal; 0, central; 1, inner. 2, outer; RNFL, retina nerve fiber layer; GCL, ganglion cell layer.

## References

[1] Netter . Atlas Human Anatomy. 8th. Barcelona: Elsevier (2023).

[2] Rosseau GL . Pituitary tumors and transsphenoidal surgery. Dis Mon. (2011) 57:607–14. doi: 10.1016/j.disamonth.2011.08.003 22036116

[3] Wang MTM Meyer JA Danesh-Meyer HV . Neuro-ophthalmic evaluation and management of pituitary disease. Eye (Lond). (2024) 38:2279–88. doi: 10.1038/s41433-024-03187-x 39039214 PMC11306754

[4] Kopczak A Renner U Stalla GK . Advances in understanding pituitary tumors. F1000Prime Rep. (2014) 6:5. doi: 10.12703/p6-5 24592317 PMC3883424

[5] Villwock JA Villwock M Deshaies E Goyal P . Significant increases of pituitary tumors and resections from 1993 to 2011. Int Forum Allergy Rhinol. (2014) 4:767–70. doi: 10.1002/alr.21356 25145472

[6] Mendes D Vydalie D Abdellaoui PAT Fiqhi PAA Mouzari P Oubaaz P . Pituitary adenoma revealed by optic neuropathy: a case report and literature review. Scholars J Med Case Rep. (2022) 10:1131–5. doi: 10.36347/sjmcr.2022.v10i11.017

[7] Lee CH . Pituitary neuroendocrine tumor: Is it benign or Malignant? Brain Tumor Res Treat. (2023) 11:173. doi: 10.14791/btrt.2023.0015 37550816 PMC10409618

[8] Geevarghese A Wollstein G Ishikawa H Schuman JS . Optical coherence tomography and glaucoma. Annu Rev Vis Sci. (2021) 7:693–726. doi: 10.1146/annurev-vision-100419-111350 34242054 PMC9184968

[9] Yang N Zhu H Ma J Shao Q . OCT and OCTA in dysthyroid optic neuropathy: a systematic review and meta-analysis. BMJ Open Ophthalmol. (2023) 8:e001379. doi: 10.1136/bmjophth-2023-001379 37996119 PMC10668299

[10] Smeets F Margot A Barbosa-Breda J Stalmans I Lemmens S . Differentiating ischemic optic neuropathy from glaucoma using diagnostic tests. Ophthalmic Res. (2024) 67:154–71. doi: 10.1159/000535568 38262372

[11] Sekhri R Kuht HJ Tu Z Maconachie GDE Shenoy R Prakash E . Identifying biomarkers for papilledema and pseudopapilledema. Sci Rep. (2025) 15:24847. doi: 10.1038/s41598-025-09778-2 40640273 PMC12246050

[12] Ito M Suda K Nakano E Tagawa M Miyata M Kashii S . Influence of tumor characteristics on visual field outcomes after pituitary adenoma surgery. J Neuroophthalmol. (2023) 43:376–82. doi: 10.1097/wno.0000000000001735 36730898

[13] Han KE Choi H Kim SJ Lee SM Lee JE . Clinical efficacy of optical coherence tomography parameters to predict the visual field outcome following pituitary adenoma surgery. PloS One. (2024) 19:e0313521. doi: 10.1371/journal.pone.0313521 39531448 PMC11556729

[14] Chung YS Na M Yoo J Kim W Jung IH Moon JH . Optical coherent tomography predicts long-term visual outcome of pituitary adenoma surgery: New perspectives from a 5-year follow-up study. Neurosurgery. (2020) 88:106–12. doi: 10.14791/btrt.2022.10.f-1212 32735666

[15] Chung YS Na M Yoo J Kim W Jung IH Moon JH . Optical coherent tomography predicts long-term visual outcome of pituitary adenoma surgery: New perspectives from a 5-year follow-up study. Neurosurgery. (2020) 88:106–12. doi: 10.14791/btrt.2022.10.f-1212 32735666

[16] Duru N Ersoy R Altinkaynak H Duru Z Çağil N Çakir B . Evaluation of retinal nerve fiber layer thickness in acromegalic patients using spectral-domain optical coherence tomography. Semin Ophthalmol. (2016) 31:285–90. doi: 10.3109/08820538.2014.962165 25380485

[17] Wang JW . Clinical applications of somatostatin analogs for growth hormone-secreting pituitary adenomas. Patient Prefer Adherence. (2024) 8:43–51. doi: 10.2147/ppa.s53930 24421637 PMC3888346

[18] Paul DA Rodrigue A Contento N Haber S Hoang R Rahmani R . Prolactin at moderately increased levels confers a neuroprotective effect in non-secreting pituitary macroadenomas. PloS One. (2022) 17:e0271690. doi: 10.1371/journal.pone.0271690. PMID: 35921360. 35921360 PMC9348739

[19] Shimon I Sosa E Mendoza V Greenman Y Tirosh A Espinosa E . Giant prolactinomas larger than 60 mm in size: a cohort of massive and aggressive prolactin-secreting pituitary adenomas. Pituitary. (2016) 19:429–36. doi: 10.1159/000495184 27138902

[20] Arroba AI Campos-Caro A Aguilar-Diosdado M Valverde ÁM . IGF-1, inflammation and retinal degeneration: a close network. Front Aging Neurosci. (2018) 10:203. doi: 10.3389/fnagi.2018.00203 30026694 PMC6041402

[21] Kaštelan S Orešković I Bišćan F Kaštelan H Gverović Antunica A . Inflammatory and angiogenic biomarkers in diabetic retinopathy. Biochem Med (Zagreb). (2020) 30:030502. doi: 10.11613/BM.2020.030502 32774120 PMC7394255

[22] Cakir B Hellström W Tomita Y Fu Z Liegl R Winberg A . IGF1, serum glucose, and retinopathy of prematurity in extremely preterm infants. JCI Insight. (2020) 5:e140363. doi: 10.1172/jci.insight.140363 33004691 PMC7566718

[23] Triebel J Bertsch T Clapp C . Prolactin and vasoinhibin are endogenous players in diabetic retinopathy revisited. Front Endocrinol (Lausanne). (2022) 13:994898. doi: 10.3389/fendo.2022.994898 36157442 PMC9500238

[24] Di Somma C Scarano E de Alteriis G Barrea L Riccio E Arianna R . Is there any gender difference in epidemiology, clinical presentation and co-morbidities of non-functioning pituitary adenomas? A prospective survey of a National Referral Center and review of the literature. J Endocrinol Invest. (2021) 44:957–68. doi: 10.1007/s40618-020-01379-2 32894472

[25] Dzialach L Sobolewska J Zak Z Respondek W Witek P . Prolactin-secreting pituitary adenomas: male-specific differences in pathogenesis, clinical presentation and treatment. Front Endocrinol (Lausanne). (2024) 15:1338345. doi: 10.3389/fendo.2024.1338345 38370355 PMC10870150

[26] Colao A Sarno AD Cappabianca P Briganti F Pivonello R Somma CD . Gender differences in prevalence, clinical features and response to cabergoline in hyperprolactinemia. Eur J Endocrinol. (2003) 148:325–31. doi: 10.1530/eje.0.1480325 12611613

[27] Hudson H Rissell C Gauderman WJ Feldon SE . Pituitary tumor volume as a predictor of postoperative visual field recovery. Quantitative analysis using automated static perimetry and computed tomography morphometry. J Clin Neuroophthalmol. (1991) 11:280–3. 1838550

[28] Lee JP Park IW Chung YS . The volume of tumor mass and visual field defect in patients with pituitary macroadenoma. Korean J Ophthalmol. (2011) 25:37–41. doi: 10.3341/kjo.2011.25.1.37 21350693 PMC3039193

[29] Hernández-Echevarría O Cuétara-Lugo EB Pérez-Benítez MJ González-Gómez JC González-Diez HR Mendoza-Santiesteban CE . Bi-nasal sectors of ganglion cells complex and visual evoked potential amplitudes as biomarkers in pituitary macroadenoma management. Front Integr Neurosci. (2022) 16. doi: 10.3389/fnint.2022.1034705 PMC9730037 36506477

[30] Hernández-Echevarría O Cuétara-Lugo EB Pérez-Benítez MJ González-Gómez JC González-Diez HR Mendoza-Santiesteban CE . Bi-nasal sectors of ganglion cells complex and visual evoked potential amplitudes as biomarkers in pituitary macroadenoma management. Front Integr Neurosci. (2022) 16:1034705. doi: 10.3389/fnint.2022.1034705 36506477 PMC9730037

[31] Pelsma ICM Verstegen MJT de Vries F Notting IC Broekman MLD van Furth WR . Quality of care evaluation in non-functioning pituitary adenoma with chiasm compression: visual outcomes and timing of intervention clinical recommendations based on a systematic literature review and cohort study. Pituitary. (2020) 23:417–29. doi: 10.1007/s11102-020-01044-0 32419072 PMC7316692

[32] Khadamy J . The role of optical coherence tomography in the early detection of chiasmal compression: Hypophyseal adenoma presenting with bitemporal nerve fiber layer thinning. Cureus. (2024) 16:e55371. doi: 10.7759/cureus.55371 38562328 PMC10982832

[33] Carbonelli M La Morgia C Savini G Cascavilla ML Borrelli E Chicani F . Macular microcysts in mitochondrial optic neuropathies: Prevalence and retinal layer thickness measurements. PloS One. (2015) 10:e0127906. doi: 10.1371/journal.pone.0127906 26047507 PMC4457906

[34] Kaushik M Wang CY Barnett MH Garrick R Parratt J Graham SL . Inner nuclear layer thickening is inversley proportional to retinal ganglion cell loss in optic neuritis. PloS One. (2013) 8:e78341. doi: 10.1371/journal.pone.0078341 24098599 PMC3789678

[35] Saidha S Sotirchos ES Ibrahim MA Crainiceanu CM Gelfand JM Sepah YJ . Microcystic macular oedema, thickness of the inner nuclear layer of the retina, and disease characteristics in multiple sclerosis: a retrospective study. Lancet Neurol. (2012) 11:963–72. doi: 10.1016/s1474-4422(12)70213-2 23041237 PMC3533139

[36] Petzold A Balcer LJ Calabresi PA Costello F Frohman TC Frohman EM . Retinal layer segmentation in multiple sclerosis: a systematic review and meta-analysis. Lancet Neurol. (2017) 16:797–812. doi: 10.1016/s1474-4422(17)30278-8 28920886

[37] Park G Khan MH Andrushko JW Banaj N Borich MR Boyd LA . Associations between contralesional neuroplasticity and motor impairment through deep learning-derived MRI regional brain age in chronic stroke (ENIGMA): a multicohort. retrospective. observational study. Lancet Digital Health. (2026) 8:100942. doi: 10.1016/j.landig.2025.100942 41577565 PMC12949600

[38] Parisi V Ziccardi L Tanga L Barbano L Tinelli E Coppola G . Citicoline oral solution induces functional enhancement and synaptic plasticity in patients with open-angle glaucoma. J Clin Med. (2025) 15:223. doi: 10.3390/jcm15010223 41517474 PMC12786903

[39] Qiu Y Zhang Q Tang J Cheng Y Wang Y Wang Z . Synaptic control of retinal ganglion cell survival and axon regeneration. Mol Neurodegener. (2026) 21:15. doi: 10.1186/s13024-026-00929-1 41606681 PMC12924416

[40] Sydnor VJ Bagautdinova J Larsen B Arcaro MJ Barch DM Bassett DS . Human thalamocortical structural connectivity develops in line with a hierarchical axis of cortical plasticity. Nat Neurosci. (2025) 28:1772–86. doi: 10.1038/s41593-025-01991-6 40615590 PMC12321582

[41] Cordeiro MF Hill D Patel R Corazza P Maddison J Younis S . Detecting retinal cell stress and apoptosis with DARC: Progression from lab to clinic. Prog Retin Eye Res. (2022) 86:100976. doi: 10.1016/j.preteyeres.2021.100976 34102318

[42] Miyagishima KJ Nadal-Nicolás FM Ma W Li W . Annexin-V binds subpopulation of immune cells altering its interpretation as an *in vivo* biomarker for apoptosis in the retina. Int J Biol Sci. (2024) 20:6073–89. doi: 10.7150/ijbs.102551 39664578 PMC11628321

[43] Parikh RS Parikh SR Sekhar GC Prabakaran S Babu JG Thomas R . Normal age-related decay of retinal nerve fiber layer thickness. Ophthalmology. (2007) 114:921–6. doi: 10.1016/j.ophtha.2007.01.023 17467529

[44] Celebi ARC Mirza GE . Age-related change in retinal nerve fiber layer thickness measured with spectral domain optical coherence tomography. Invest Ophthalmol Vis Sci. (2013) 54:8095–103. doi: 10.1167/iovs.13-12634 24194190

[45] Frara S Rodriguez-Carnero G Formenti AM Martinez-Olmos MA Giustina A Casanueva FF . Pituitary tumors centers of excellence. Endocrinol Metab Clinics North America. (2020) 49:553–64. doi: 10.1016/j.ecl.2020.05.010 32741488

[46] Okrent Smolar AL Ray HJ Dattilo M Bouthour W Berman G Peragallo JH . Neuro-ophthalmology emergency department and inpatient consultations at a large academic referral center. Ophthalmology. (2023) 130:1304–12. doi: 10.1016/j.ophtha.2023.07.028 37544433

