# Supplementary material for: Neuroretinal damage associated with pituitary macroadenoma: endocrine and radiological predictors and correlation with optical coherence tomography-derived biomarkers
Source: Front Endocrinol (Lausanne). 2026 Jun 26;17:1829465. doi: 10.3389/fendo.2026.1829465 (PMC13350339; doi:10.3389/fendo.2026.1829465)
Supplement: Supplementary Material 2 — Hormone values in patients with a pituitary tumor. The normal value range is shaded in green. TSH, Thyroid-stimulating hormone. T4, Tetraiodothyronine/Thyroxine. LH, Luteinizing Hormone. FSH, Follicle-stimulating hormone. ACTH, Adrenocorticotropic hormone. GH, Growth hormone. IGF1, Insulin-like growth factor 1. [file Supplementaryfile1.docx]

SUPPLEMENTARY FILE

| Hormone | Normal Values (Ranges) |
| --- | --- |
| **TSH** | 0.38–5.33 µUI/L |
| **T4** | 0.58–1.64 ng/dL |
| **LH** | M: 1.26–10.05 mUI/mL  F: 1.20–58.64 mUI/mL |
| **FSH** | M: 1.27–19.26 mUI/mL  F: 2.74–26.72 mUI/mL |
| **Prolactin** | M: 2.64–13.13 ng/mL  F: 2.74–26.72 ng/mL |
| **ACTH** | 7.2–63.3 pg/mL |
| **Cortisol** | 5–25 µg/dL |
| **GH** | 0.01–3.607 ng/dL |
| **IGF-1** | 53–215 ng/mL |

***Supplementary Material 1****: Normal hormonal reference ranges. Abbreviations: M, males; F, females; TSH, thyroid-stimulating hormone; T4, tetraiodothyronine/thyroxine; LH, luteinizing hormone; FSH, follicle-stimulating hormone; ACTH, adrenocorticotropic hormone; GH, growth hormone; IGF-1, insulin-like growth factor 1*


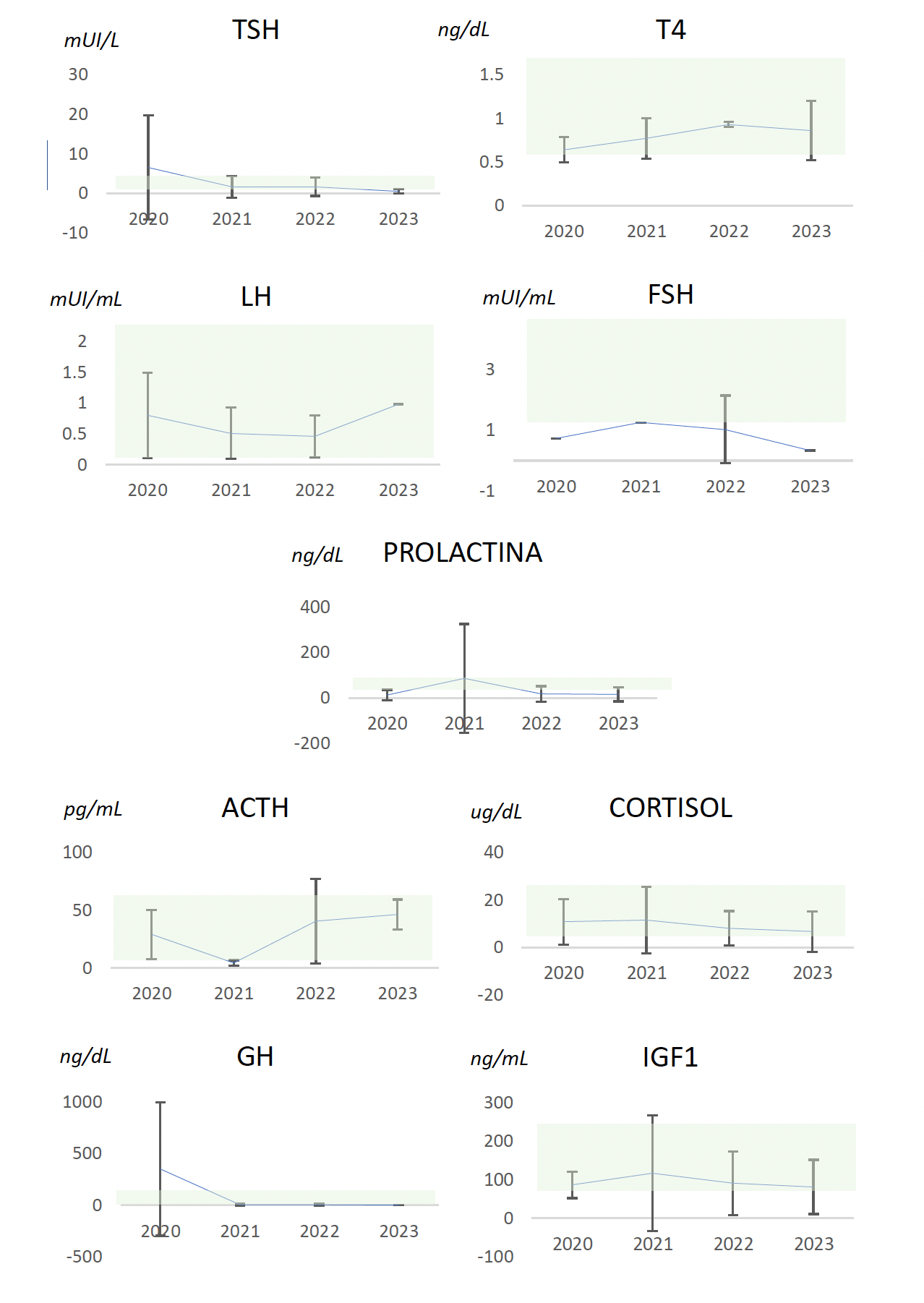


***Supplementary Material 2****:* *Hormone values in patients with a pituitary tumor. The normal value range is shaded in green. Abbreviations: TSH: Thyroid-stimulating hormone. T4: Tetraiodothyronine / Thyroxine. LH: Luteinizing Hormone. FSH: Follicle-stimulating hormone. ACTH: Adrenocorticotropic hormone. GH: Growth hormone. IGF1: Insulin-like growth factor 1.*

***Supplementary Material 3 for Figure 6****. Structural neuroretinal changes detected by optical coherence tomography (OCT) in patients with pituitary macroadenoma. Macular and peripapillary sectors are color-coded where thickness decreased (p < 0.05).*

*Green, tumor duration > 5 years Pink, nonfunctioning tumor; Red, normal hormone levels; Blue, tumor > 10,000 mm³; Orange, surgery; Purple, relapse. Abbreviations: GCL, ganglion cell layer; IPL, inner plexiform layer; INL, inner nuclear layer; RNFL, retinal nerve fiber layer; OPL, outer plexiform layer; ONL, outer nuclear layer; RPE, retinal pigment epithelium. m, macular sectors: S1, superior inner; S2, superior outer; I1, inferior inner; I2, inferior outer; N1, nasal inner; N2, nasal outer; T1, temporal inner; T2, temporal outer. p, peripapillary sectors: NS, nasal–superior; N, nasal; NI, nasal–inferior; TI, temporal–inferior; T, temporal; TS, temporal–superior.*

| **Ophthalmic parameters** | **Pre-surgery** | **Post-surgery** | **p** |
| --- | --- | --- | --- |
| RETINA Mean C0 µm | 252.58 ± 120.09 | 275.55 ± 39.87 | 0.218 |
| RETINA Mean N1 µm | 283.41 ± 121.12 | 320.64 ± 29.02 | 0.419 |
| RETINA Mean N2 µm | 263.58 ± 110.62 | 295.57 ± 24.19 | 0.719 |
| RETINA Mean S1 µm | 289.13 ± 125.18 | 326.94 ± 37.64 | 0.470 |
| RETINA Mean S2 µm | 253.27 ± 105.60 | 290.39 ± 21.03 | 0.769 |
| RETINA Mean T1 µm | 283.03 ± 121.80 | 318.85 ± 27.15 | 0.865 |
| RETINA Mean T2 µm | 237.84 ± 98.50 | 275.66 ± 19.21 | 0.880 |
| RETINA Mean I1 µm | 211.39 ± 158.85 | 316.53 ± 21.80 | 0.699 |
| RETINA MeanI2 µm | 245.31 ± 102.08 | 275.66 ± 21.69 | 0.764 |
| RETINA Center µm | 239.50 ± 124.03 | 242.05 ± 42.46 | 0.212 |
| RNFL Mean C0 µm | 13.61 ± 9.53 | 11.47 ± 6.93 | 0.522 |
| RNFL Mean N1 µm | 19.92 ± 5.36 | 18.06 ± 7.62 | 0.686 |
| RNFL Mean N2 µm | 39.04 ± 13.50 | 34.24 ± 13.44 | 0.998 |
| RNFL Mean S1 µm | 21.64 ± 8.58 | 19.56 ± 8.82 | 0.103 |
| RNFL Mean S2 µm | 32.64 ± 6.99 | 29.18 ± 9.54 | 0.595 |
| RNFL Mean T1 µm | 19.50 ± 7.36 | 18.33 ± 10.45 | 0.653 |
| RNFL Mean T2 µm | 18.96 ± 2.02 | 17.86 ± 5.06 | 0.214 |
| RNFL Mean I1 µm | 24.05 ± 4.96 | 22.31 ± 6.88 | 0.075 |
| RNFL Mean I2 µm | 37.05 ± 8.48 | 35.36 ± 12.81 | 0.833 |
| RNFL Center µm | 6.66 ± 18.71 | 3.68 ± 5.32 | **0.033** |
| GCL Mean C0 µm | 16.25 ± 9.87 | 13.46 ± 4.52 | **<0.001** |
| GCL Mean N1 µm | 36.89 ± 11.78 | 32.91 ± 8.09 | **<0.001** |
| GCL Mean N2 µm | 31.51 ± 5.83 | 29.08 ± 5.03 | **<0.001** |
| GCL Mean S1 µm | 42.05 ± 11.07 | 36.46 ± 7.38 | **0.005** |
| GCL Mean S2 µm | 31.27 ± 4.98 | 30.21 ± 4.49 | **<0.001** |
| GCL Mean T1 µm | 40.69 ± 7.52 | 37.58 ± 6.19 | 0.209 |
| GCL Mean T2 µm | 32.78 ± 5.23 | 31.75 ± 4.65 | **<0.001** |
| GCL Mean I1 µm | 40.96 ± 10.02 | 39.21 ± 7.65 | **<0.001** |
| GCL Mean I2 µm | 29.95 ± 4.77 | 27.58 ± 3.50 | **0.020** |
| GCL Center µm | 7.30 ± 10.49 | 5.19 ± 4.40 | **0.018** |

***Supplementary Material 4 for Figure 8:*** *Abbreviations: S: superior; T: temporal; I: inferior; N: nasal; 0: central; 1: inner. 2: outer; RNFL: retina nerve fiber layer; GCL: ganglion cell layer.*
